# Methanol Extract of *Artemisia apiacea* Hance Attenuates the Expression of Inflammatory Mediators via NF-*****κ*****B Inactivation

**DOI:** 10.1155/2013/494681

**Published:** 2013-10-22

**Authors:** Ji Choul Ryu, Sang Mi Park, Min Hwangbo, Sung Hui Byun, Sae Kwang Ku, Young Woo Kim, Sang Chan Kim, Seon Young Jee, Il Je Cho

**Affiliations:** ^1^Department of Ophthalmology, Otolaryngology & Dermatology, College of Korean Medicine, Daegu Haany University, Gyeongsangbuk-do 712-715, Republic of Korea; ^2^Medical Research Center for Globalization of Herbal Formulation, College of Korean Medicine, Daegu Haany University, Gyeongsangbuk-do 712-715, Republic of Korea; ^3^College of Korean Medicine, Daegu Haany University, Gyeongsangbuk-do 712-715, Republic of Korea

## Abstract

*Artemisia apiacea* Hance is one of the most widely used herbs for the treatment of malaria, jaundice, and dyspeptic complaint in oriental medicine. This study investigated the effects of methanol extracts of *A. apiacea* Hance (MEAH) on the induction of inducible nitric oxide synthase (iNOS) and proinflammatory mediators by lipopolysaccharide (LPS) in Raw264.7 macrophage cells and also evaluated the *in vivo* effect of MEAH on carrageenan-induced paw edema in rats. MEAH treatment in Raw264.7 cells significantly decreased LPS-inducible nitric oxide production and the expression of iNOS in a concentration-dependent manner, while MEAH (up to 100 **μ**g/mL) had no cytotoxic activity. Results from immunoblot analyses and ELISA revealed that MEAH significantly inhibited the expression of cyclooxygenase-2, tumor necrosis factor-*α*, interleukin-1*β*, and interleukin-6 in LPS-activated cells. As a plausible molecular mechanism, increased degradation and phosphorylation of inhibitory-*κ*B*α* and nuclear factor-*κ*B accumulation in the nucleus by LPS were partly blocked by MEAH treatment. Finally, MEAH treatment decreased the carrageenan-induced formation of paw edema and infiltration of inflammatory cells in rats. These results demonstrate that MEAH has an anti-inflammatory therapeutic potential that may result from the inhibition of nuclear factor-*κ*B activation, subsequently decreasing the expression of proinflammatory mediators.

## 1. Introduction 

Inflammation is regarded as a protective response against tissue injury or destruction. During the inflammatory process, a number of inflammatory cells infiltrate into the damaged tissue and produce inflammatory mediators that exaggerate inflammatory responses characterized by redness, swelling, fever, and pain [1]. Nitric oxide (NO), prostanoids, tumor necrosis factor-*α* (TNF-*α*), and interleukins (ILs) produced from infiltrated cells or damaged tissue act as pleiotropic effector molecules to amplify acute inflammation and lead to the activation of adaptive immune response. On the other hand, inappropriate inflammation plays a pivotal role in the pathogenesis of a variety of disorders. Thus, it is essential that the inflammatory process must be controlled spaciotemporally to prevent unwanted tissue damage and reduce inflammation-related disorders.

A variety of signaling pathways are involved in transducing the inflammatory response. Among them, nuclear factor-kappa B (NF-*κ*B) is an essential signaling molecule to increase the production of effector molecules related to acceleration of the inflammatory process [2]. The activity of NF-*κ*B is largely dependent on its interaction with inhibitory-*κ*B (I-*κ*B) protein in the cytoplasm. Inflammatory stimuli trigger I-*κ*B phosphorylation through I-*κ*B kinase and degradation via the ubiquitin-proteasome system and the subsequent dissociation of the NF-*κ*B complex from I-*κ*B [2, 3]. Activation of NF-*κ*B in the cytoplasm allows it to translocate into the nucleus, where it binds to the promoter region of target genes that are associated with immune responses, cell survival, apoptosis, and so on [4]. 

Because they have relatively fewer side effects, Herbal medications have garnered wide interest as complementary and alternative therapeutics and are becoming more popular. Moreover, novel therapeutic candidates have been screened from plentiful sources of medicinal herbs to manage inappropriate immune responses and developed as anti-inflammatory drugs over the last few decades. To expand the usage of herbal medications, the pharmacological efficacy of medicinal herbs should be evaluated both *in vitro* and *in vivo*, and their molecular mechanisms need to be clarified. Lipopolysaccharide (LPS)-mediated macrophage activation using Raw264.7 cells is a well-established *in vitro* inflammation model [5, 6]. Moreover, carrageenan-induced paw edema formation is regarded as a representative acute inflammation *in vivo* model [7]. Therefore, both combined models have been extensively studied to examine the anti-inflammatory effect of medicinal herbs and to identify the putative molecular mechanism [5, 6, 8]. 

*Artemisia apiacea* Hance is one of the most widely used herbs in oriental medicine [9]. Artemisin found in *A. apiacea* Hance is not only a standard treatment worldwide for falciparum malaria but also has a number of pharmacological effects involving anticancer, antiviral, and immunosuppressive activities [10, 11]. Regardless of a number of studies about artemisin and its derivatives, the pharmacological effect of *A. apiacea* Hance itself, the parental herb containing artemisin, has not been fully understood, and little is known about the anti-inflammatory effect of the herb.

This study examined the effect of methanol extracts of *A. apiacea* Hance (MEAH) on the expression of inflammatory mediators in LPS-stimulated Raw264.7 cells and also investigated the *in vivo* effect of MEAH on carrageenan-induced edema formation. With the data, this study was extended to explore the effects of MEAH on the NF-*κ*B signaling pathway as a plausible molecular mechanism. 

## 2. Materials and Methods 

### 2.1. MEAH Preparation and Reagents

*A. apiacea* Hance was purchased from Daewon Pharmacy (Daegu, Republic of Korea). MEAH was prepared by extracting 400 g of *A. apiacea* Hance in 3 L of 100% methanol for 48 h. The MEAH was filtered through a 0.2 *μ*m filter (Nalgene, NY, USA), lyophilized by a vacuum evaporator, and stored at −20°C until use. The amount of MEAH was estimated from the dry weight of lyophilized MEAH. The yield of lyophilized MEAH was 6.03%. Antibody directed phosphorylated inhibitory-*κ*B*α* (p-I-*κ*B*α*), lamin A/C, and horseradish peroxidase-conjugated secondary antibodies were obtained from Cell Signaling Biotechnology (Beverly, MA, USA). Anti-iNOS and anti-COX-2 antibodies were supplied from BD Bioscience (San Jose, CA, USA). Anti-I-*κ*B*α*, anti-NF-*κ*B (p65 subunit), and anti-actin antibodies were purchased from Santa Cruz Biotechnology (Santa Cruz, CA, USA). Polyethylene glycol no. 400 (PEG) was obtained from Yakury Pure Chemical Co. (Kyoto, Japan). Carrageenan, dexamethasone, LPS, 3-(4,5-dimethyl-2-thiazolyl)-2,5-diphenyl-2H-tetrazolium bromide (MTT), and other reagents were purchased from Sigma Chemical Co. (St. Louis, MO, USA).

### 2.2. Cell Culture

 Raw264.7 cells, a murine macrophage cell line, were obtained from American Type Culture Collection (Rockville, MD, USA). The cells were maintained in Dulbecco's modified Eagle's medium (DMEM) containing 10% fetal bovine serum, 50 U/mL penicillin, and 50 *μ*g/mL streptomycin at 37°C in a humidified atmosphere with 5% CO_2_. For all experiments, the cells were grown to 80–90% confluency and were subjected to no more than 20 cell passages. The Raw264.7 cells were incubated in medium without fetal bovine serum for 12 h and then subsequently exposed to 1 *μ*g/mL LPS for the indicated time periods (1–24 h). MEAH, dissolved in dimethyl sulfoxide, was added to the incubation medium 1 h prior to the addition of LPS.

### 2.3. Cell Viability Assay

The cells were plated at a density of 5 × 10^4^ cells per well in 96-well plates to examine the cytotoxicity of MEAH. Cells were serum starved for 12 h and then treated with MEAH in the presence or absence of 1 *μ*g/mL LPS for the next 24 h. After incubation of the cells, viable cells were stained with MTT (0.5 mg/mL, 4 h) according to the previous report [5, 6]. 

### 2.4. Assay of Nitrite Production

NO production was monitored by measuring the nitrite content in culture medium [5, 6]. Samples were mixed with Griess reagent (Sigma, St. Louis, MO, USA), and absorbance was measured at 540 nm.

### 2.5. Enzyme-Linked Immunosorbent Assay (ELISA)

 Raw264.7 cells were preincubated with MEAH for 1 h and continuously exposed to LPS for 18 h. TNF-*α*, IL-1*β*, IL-6, and prostaglandin E_2_ (PGE_2_) production in the medium was measured by ELISA using each antibody and biotinylated secondary antibody according to the manufacturer's instructions (Pierce, Woburn, MA, USA).

### 2.6. Sample Preparation and Immunoblot Analysis

 Whole cell lysates and nuclear extracts were prepared according to previously established methods [5, 6]. Briefly, cells were lysed in buffer containing 20 mM Tris-HCl (pH 7.5), 1% Triton X-100, 137 mM sodium chloride, 10% glycerol, 2 mM EDTA, 1 mM sodium orthovanadate, 25 mM *β*-glycerophosphate, 2 mM sodium pyrophosphate, 1 mM phenylmethylsulfonyl fluoride, and 1 *μ*g/mL leupeptin. Cell lysates were collected by centrifugation at 10,000 ×g for 10 min. To prepare nuclear extracts, cells were allowed to swell in 100 *μ*L of lysis buffer (10 mM HEPES (pH 7.9), 10 mM KCl, 0.1 mM EDTA, 0.5% Nonidet-P40, 1 mM dithiothreitol and 0.5 mM phenylmethylsulfonyl fluoride). After centrifugation of the samples, the pellets containing crude nuclei were resuspended in 50 *μ*L of extraction buffer containing 20 mM HEPES (pH 7.9), 400 mM NaCl, 1 mM EDTA, 1 mM dithiothreitol, and 1 mM phenylmethylsulfonyl fluoride and incubated for 30 min on ice. The samples were centrifuged at 15,800 ×g for 10 min to obtain the supernatant containing nuclear extracts. Protein contents of samples were measured using BCA assay (Pierce, Woburn, MA, USA). Equal amounts of protein were resolved by sodium dodecyl sulfate-polyacrylamide gel electrophoresis and then transferred to nitrocellulose membranes (Schleicher & Schuell GmbH, Dassel, Germany). Immunoreactive proteins of interest were visualized by an ECL chemiluminescence detection kit (Amersham Biosciences, Buckinghamshire, UK). Equal protein loading among the samples in each gel was verified by immunoblotting with an antibody directed against actin or lamin A/C.

### 2.7. Carrageenan-Induced Paw Edema

 Animal studies were conducted in accordance with the institutional guidelines of Daegu Haany University for the care and use of laboratory animals. Sprague-Dawley rats at 6 weeks of age (male, 140–160 g) were provided from Samtako Co. (Osan, Republic of Korea), acclimatized for 1 week, and maintained in a clean room at the Animal Center of the College of Korean Medicine, Daegu Haany University. Animals were caged under the supply of filtered pathogen-free air, commercial rat chow (Purina, Republic of Korea), and water *ad libitum* at a temperature between 20°C and 23°C with 12 h light and dark cycles and relative humidity of 50%. Rats (*N* = 20) were randomly divided into four groups, and each group consisted of five animals. MEAH, dissolved in 40% PEG, was orally administered to rats at a dose of 1.0 or 0.3 g/kg/day for 3 consecutive days. Dexamethasone (1 mg/kg/day, p.o.), a representative anti-inflammatory drug, was used as a positive control [6]. To induce acute phase paw inflammation, rats were injected subcutaneously into the right hind paw with a 1% solution of carrageenan dissolved in saline (0.1 mL per animal) 30 min after vehicle or drug treatment. The paw volume was measured up to 4 h after the injection at intervals of 1 h. The hind paw volume was determined volumetrically by measuring with a plethysmometer (Letica, Rochester, MI, USA). The paw edema volume was defined relative to the paw volume in carrageenan-treated rats at 0 h (i.e., paw edema volume (%) = 100 × (paw volume of treated rat at the indicated time period)/(paw volume of carrageenan-treated rat at 0 h)).

### 2.8. Histopathology

*Dorsum* and *ventrum pedis* skins were separated and fixed in 10% neutral buffered formalin, then embedded in paraffin, sectioned (3-4 *μ*m), and stained with hematoxylin and eosin. The histopathological profiles of each sample were observed under light microscope (Nikon, Japan) by certified pathologist. The thicknesses of *dorsum pedis* and *ventrum pedis* (from epidermis to dermis, keratin layers were excluded) and the number of infiltrated inflammatory cells were measured using automated image analyzer (DMI-300 Image Processing; DMI, Republic of Korea) according to the previous report with some modifications [12].

### 2.9. Statistical Analysis

 Statistical analyses were conducted using SPSS for Windows (Release 14.0 K, SPSS Inc., USA). Multiple comparison tests among different dose groups were analyzed by one-way ANOVA. The data were expressed as mean ± S.D. The criterion for statistical significance was set at *P* < 0.05 or *P* < 0.01.

## 3. Results

### 3.1. Effect of MEAH on Cell Viability and NO Production in Raw264.7 Cells

**Figure 1:**
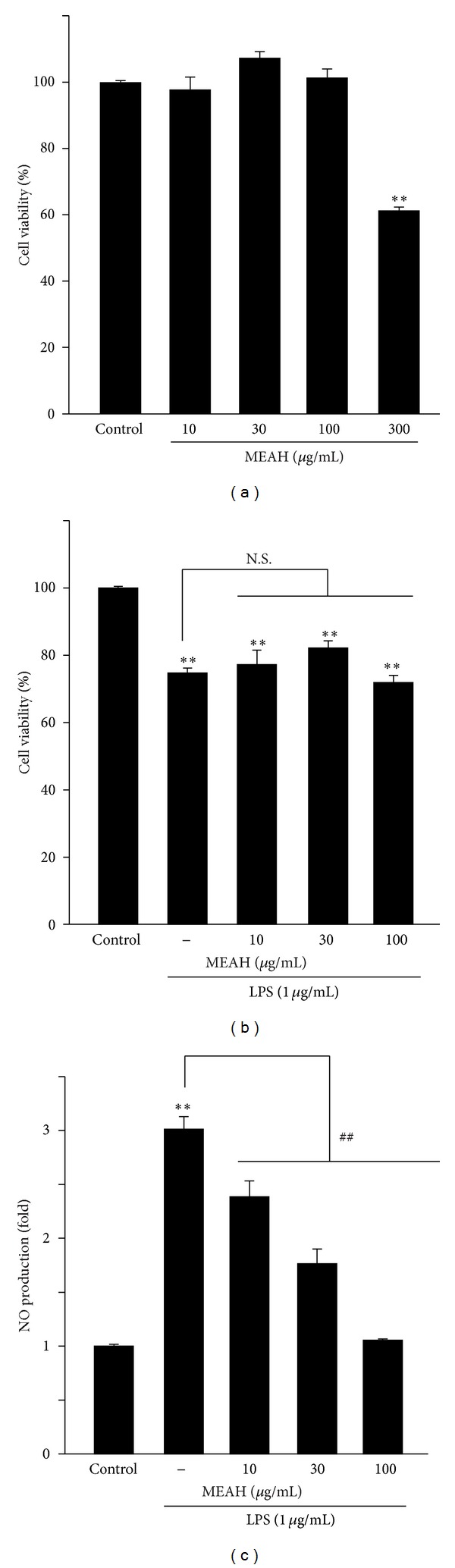
Effect of methanol extracts of *Artemisia apiacea* Hance (MEAH) on cell viability and NO production. Raw264.7 cells were incubated with 10–300 *μ*g/mL MEAH for 24 h (a) or pretreated with 10–100 *μ*g/mL MEAH for 1 h and then continuously exposed to 1 *μ*g/mL LPS for 24 h (b). After incubation, cell viability was assessed by the MTT analysis. Data represent mean ± S.D. of three separated experiments (significant as compared with vehicle-treated control cells, **P < 0.01; cell viability of control = 100%). N.S.: not significant. (c) NO production. Raw264.7 cells were pretreated with 10–100 *μ*g/mL MEAH for 1 h and then further incubated with 1 *μ*g/mL LPS for 24 h. NO release in the medium was measured by using Griess reagent. Data represent mean ± S.D. of three separated experiments (significant as compared with control cells, **P < 0.01; significant as compared with LPS-treated cells, ^##^ *P* < 0.01).

Prior to exploring the anti-inflammatory effects of MEAH in cells, any possible toxicity of MEAH was monitored by MTT analyses (Figures 1(a) and 1(b)). MTT assay indicated that treatment with MEAH up to 100 *μ*g/mL for 24 h did not show any toxicity in Raw264.7 cells. However, 300 *μ*g/mL MEAH elicited approximately 40% cell loss. As already reported, 1 *μ*g/mL LPS for 24 h inhibited cell viability slightly [13], and reduction of cell viability by LPS was not changed by pretreatment with 100 *μ*g/mL MEAH. Therefore, 10–100 *μ*g/mL MEAH was chosen for examining the anti-inflammatory effects of MEAH in subsequent experiments. Next, the effect of MEAH on NO production was examined in LPS-stimulated Raw264.7 cells. LPS (1 *μ*g/mL, 24 h) treatment increased the production of NO. However, MEAH pretreatment significantly decreased NO release in a dose-dependent manner compared with LPS-treated cells. Strong inhibition was obtained with 100 *μ*g/mL MEAH-treatment (Figure 1(c)). 

### 3.2. Effect of MEAH on Proinflammatory Mediators Production in Raw264.7 Cells

**Figure 2:**
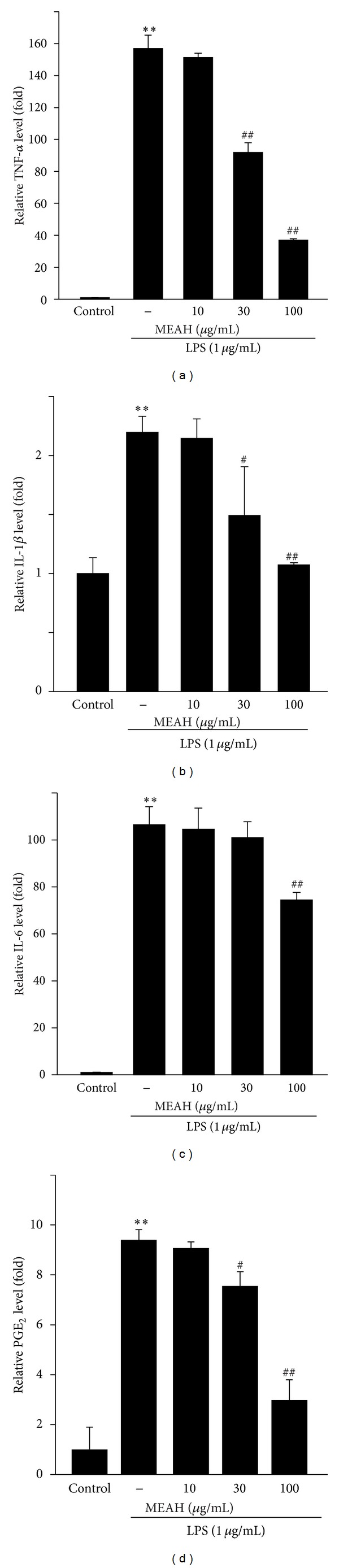
Effect of MEAH on the secretion of proinflammatory mediators in LPS-stimulated Raw264.7 cells. The cells were pretreated with 10–100 *μ*g/mL MEAH for 1 h and then continuously exposed to 1 *μ*g/mL LPS for 18 h. The levels of TNF-*α* (a), IL-1*β* (b), IL-6 (c), and PGE_2_ (d) were monitored in the medium by using ELISA. Data represent mean ± S.D. of three separated experiments (significant as compared with vehicle-treated control cells, **P < 0.01; significant as compared with LPS-treated cells, ^##^ *P* < 0.01, ^#^ *P* < 0.05; the level of proinflammatory mediators in control cells = 1).

 To investigate the effect of MEAH on the expression of proinflammatory meditators in LPS-stimulated Raw264.7 cells, the secreted levels of TNF-*α*, IL-1*β*, IL-6, and PGE_2_ were monitored in the medium by ELISA (Figures 2(a)–2(d)). As previously reported [5], LPS-treated cells (1 *μ*g/mL, for 18 h) exhibited significantly increased secretion of proinflammatory mediators (TNF-*α*, IL-1*β*, IL-6, and PGE_2_) compared with vehicle-treated cells. In contrast, MEAH pretreatment significantly blocked the secretion of TNF-*α* (Figure 2(a)), IL-1*β* (Figure 2(b)), and PGE_2_ (Figure 2(d)) in a dose-dependent manner compared with LPS-stimulated cells. In the case of IL-6, only 100 *μ*g/mL MEAH pretreatment showed a significant reduction (Figure 2(c)). These results indicate that MEAH inhibited the secretion of proinflammatory mediators in LPS-activated cells, which might result, at least in part, from the perturbation of common signaling pathways involving proinflammatory mediator induction.

### 3.3. Effect of MEAH on the Expression of Proinflammatory Enzymes in Raw264.7 Cells

**Figure 3:**
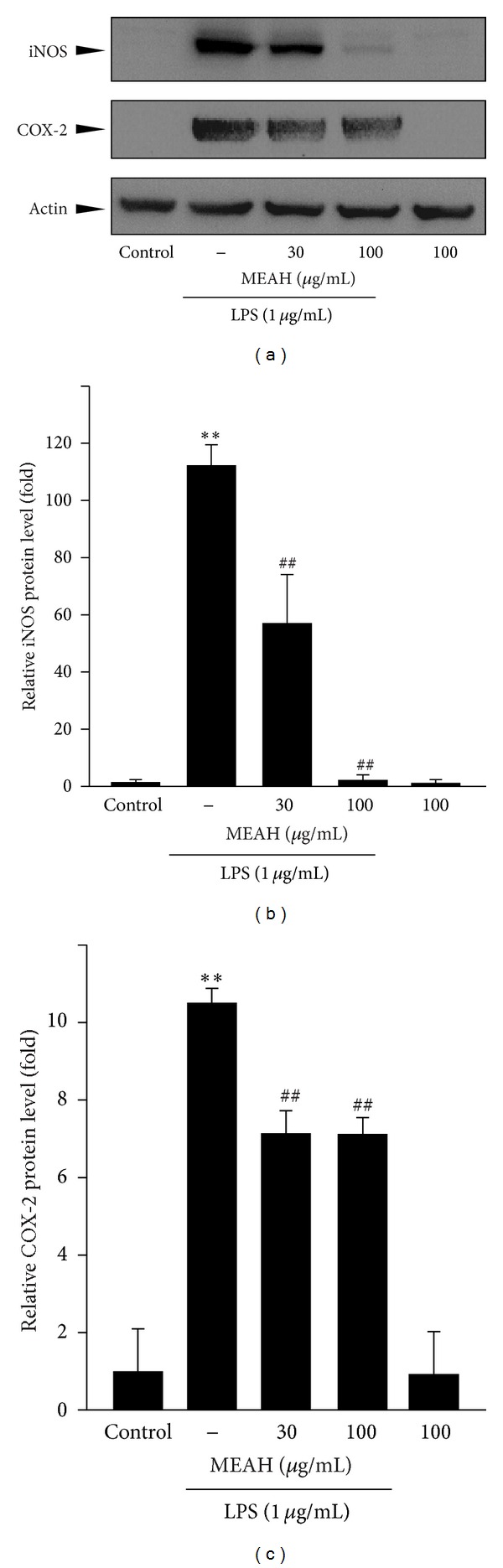
Effect of MEAH on the expression of proinflammatory enzymes in LPS-stimulated Raw264.7 cells. The cells were pretreated with 30 or 100 *μ*g/mL MEAH for 1 h and then continuously exposed to 1 *μ*g/mL LPS for 18 h. iNOS and COX-2 were immunoblotted in the cell lysates. Equal proteins loading among the samples was verified by actin immunoblotting (a). The relative iNOS and COX-2 protein levels were assessed by scanning densitometry (b, c). Data represent mean ± S.D. of three separated experiments (significant as compared with vehicle-treated control cells, **P < 0.01; significant as compared with LPS-treated cells, ^##^ *P* < 0.01; the expression level of iNOS and COX-2 in control cells = 1).

To determine whether the reduction of NO is related to the regulation of iNOS expression, immunoblot analyses against iNOS were conducted. No detectable expression of iNOS was observed in vehicle- or MEAH-treated cells, whereas LPS treatment (1 *μ*g/mL) strongly induced the expression of iNOS (Figures 3(a) and 3(b)). MEAH treatment in the presence of LPS decreased the iNOS expression in a dose-dependent manner. In particular, 100 *μ*g/mL of MEAH pretreatment almost completely prevented the iNOS induction by LPS. In our continuing effort to validate the anti-inflammatory effect of MEAH, the expression level of COX-2, the rate limiting enzyme of PG production, was further examined (Figures 3(a) and 3(c)). Like iNOS expression, no basal COX-2 expression was observed in vehicle- or MEAH-treated cells, while LPS-treated cells induced strongly the COX-2 expression. Moreover, pretreatment with MEAH slightly, but significantly, blocked the expression of COX-2. Thus, MEAH inhibited both iNOS and COX-2 expression in LPS-stimulated Raw264.7 cells.

### 3.4. Effect of MEAH on NF-*κ*B Signaling Pathway in Raw264.7 Cells

**Figure 4:**
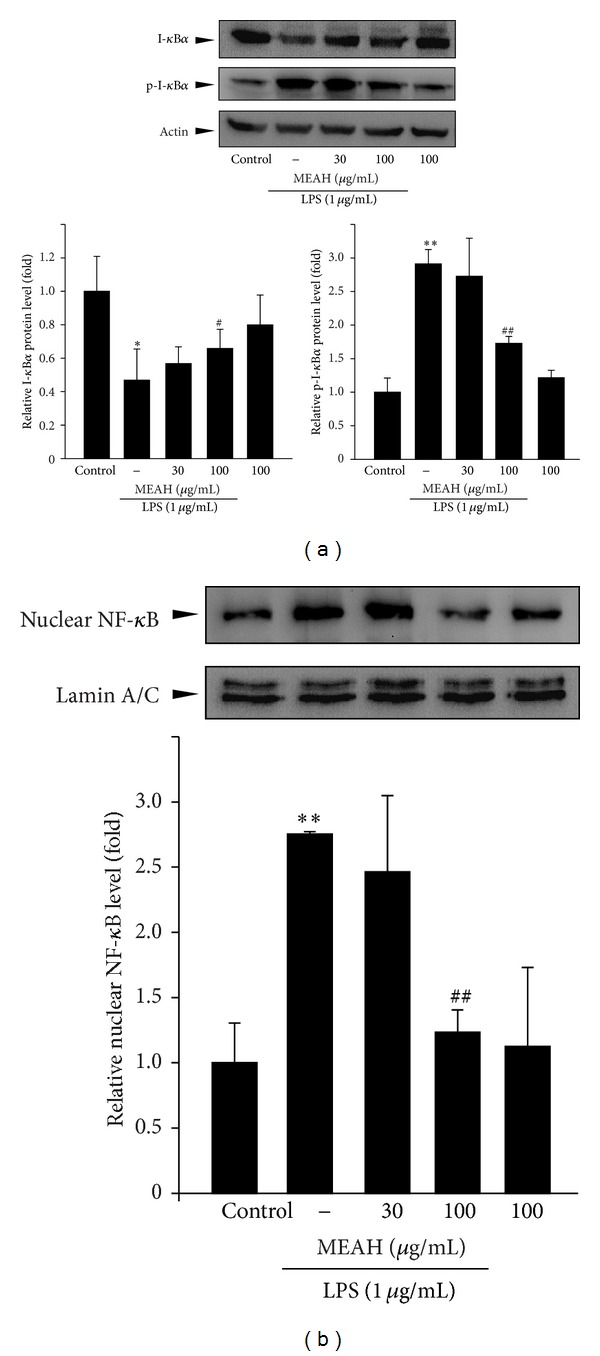
Effect of MEAH on NF-*κ*B signaling pathway in LPS-stimulated Raw264.7 cells. The cells were pretreated with 30 or 100 *μ*g/mL MEAH for 1 h and then continuously exposed to 1 *μ*g/mL LPS for 1 h. Total or phosphorylated I-*κ*B*α* in whole cell lysates (a) and nuclear NF-*κ*B (b) were assessed by immunoblotting analysis. Antibody against actin or lamin A/C was used for verifying equal protein loading of cell lysates or nuclear fractions, respectively. Data represent mean ± S.D. of three separated experiments (significant as compared with vehicle-treated control cells, **P < 0.01, *P < 0.05; significant as compared with LPS-treated cells, ^##^ *P* < 0.01, ^#^ *P* < 0.05; the expression level of each protein in control cells = 1).

 As a plausible molecular mechanism for MEAH-mediated inhibition of inflammatory response, the effect of MEAH on the NF-*κ*B signaling pathway was explored (Figure 4). LPS exposure (1 *μ*g/mL) for 1 h facilitated the degradation of I-*κ*B*α*, phosphorylation of I-*κ*B*α*, and nuclear accumulation of NF-*κ*B. However, pretreatment with 100 *μ*g/mL MEAH inhibited the degradation of I-*κ*B*α*. Furthermore, MEAH decreased the LPS-mediated I-*κ*B*α* phosphorylation and nuclear NF-*κ*B accumulation in a dose-dependent manner. Thus, the anti-inflammatory effect of MEAH might be due to inhibition of the NF-*κ*B signaling pathway.

### 3.5. Inhibitory Effect of MEAH on Carrageenan-Induced Paw Edema Formation

**Figure 5:**
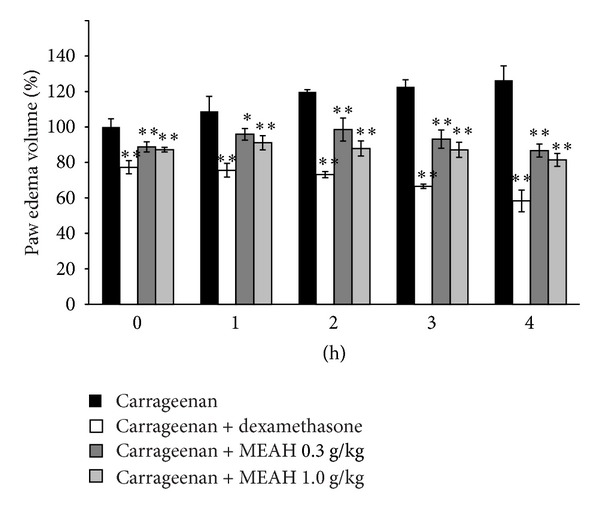
Inhibition of carrageenan-induced paw edema formation by MEAH. MEAH, dissolved in 40% PEG, was orally administered to rats at 0.3 or 1.0 g/kg/day prior to the induction of paw edema for three days. Paw edema was induced by subcutaneous injection of 1% carrageenan solution as described in methods. The swelling volume of paw was measured up to 4 h after 0.5 h carrageenan injection at intervals of 1 h by using plethysmometer. Dexamethasone (1 mg/kg, p.o.) was used as a positive drug. Data represent mean ± S.D. of five animals (significant as compared with carrageenan-treated group, **P < 0.01; *P < 0.05, paw volume at 0 h in carrageenan-treated rat = 100%).

To explore anti-inflammatory effect of MEAH *in vivo*, the carrageenan-induced paw edema model was used. Results from plethysmometer showed that formation of paw edema began to be observed as early as 1 h after a carrageenan injection. Paw swelling was increased significantly at 2 h and sustained at least up to 4 h after carrageenan injection (Figure 5). Dexamethasone pretreatment (1.0 mg/kg/day, p.o.), a positive control drug, significantly decreased edema formation. Preadministration of MEAH (1.0 or 0.3 g/kg/day) for 3 days decreased carrageenan-induced paw edema, and reduction of swelling volumes persisted for at least 4 h. Reduction of edema volume was not changed significantly among observation times.

### 3.6. Histomorphometric Effect of MEAH in Carrageenan-Induced Paw Edema

**Figure 6:**
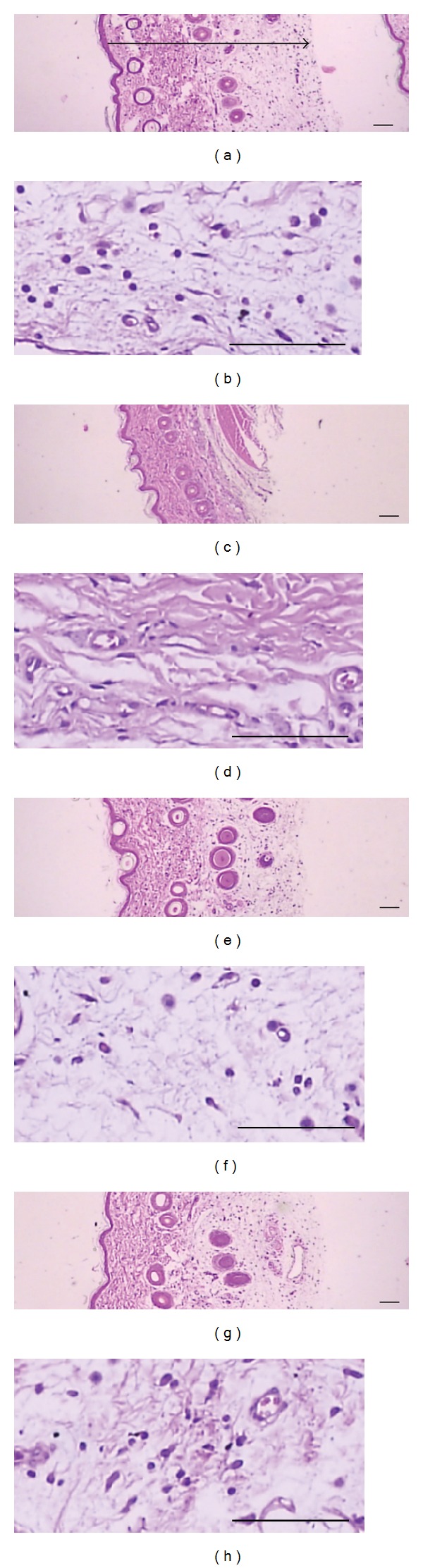
Representative histological images of the *dorsum pedis* skin in carrageenan (a, b), carrageenan + dexamethasone (c, d), carrageenan + 1.0 g/kg MEAH (e, f), and carrageenan + 0.3 g/kg MEAH (g, h) treated groups. After 4 h of carrageenan treatment, *dorsum pedis* skins were separated, and fixed in 10% neutral buffered formalin, then embedded in paraffin, sectioned and stained with hematoxylin and eosin. The arrow indicated total thickness measured (scale bars = 160 *μ*m).

**Figure 7:**
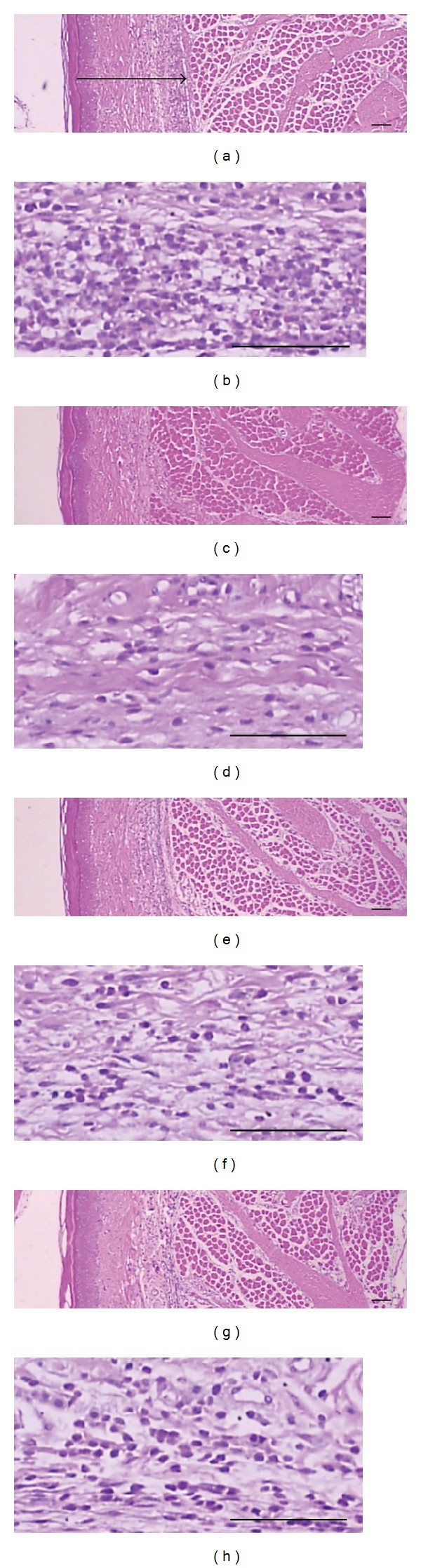
Representative histological images of the *ventrum pedis* skin in carrageenan (a, b), carrageenan + dexamethasone (c, d), carrageenan + 1.0 g/kg MEAH, (e, f), and carrageenan + 0.3 g/kg MEAH (g, h) treated groups. Tissue sections were prepared as described in Figure 6. The arrow indicated total thickness measured (scale bars = 160 *μ*m).

Histomorphometrical measurements of *dorsum* and *ventrum pedis* skin were carried out to further validate the *in vivo* anti-inflammatory effect of MEAH (Table 1). As shown in Figures 6 and 7, marked increases of the *dorsum* and *ventrum pedis* skin thicknesses were detected as results of carrageenan-induced acute edematous inflammations on carrageenan-treated rats, and marked increases of infiltrated inflammatory cells were also detected in both *dorsum* and *ventrum pedis* cutaneous regions, respectively. However, these carrageenan-induced acute edematous changes and inflammatory cell infiltrations were significantly blocked by treatment with two different doses of MEAH (Table 1). The thicknesses of *dorsum pedis* skin in 1.0 and 0.3 g/kg/day MEAH-treated groups were significantly reduced by −23.79% and −13.12% compared with carrageenan-treated rats, respectively. Moreover, the numbers of infiltrated inflammatory cells in the *dorsum pedis* skin were significantly decreased by −63.41% and −25.97% in 1.0 and 0.3 g/kg/day MEAH-treated groups, respectively (Figure 6 and Table 1). High dose of MEAH treated group (1.0 g/kg/day) further decreased both the thickness of edematous skin and the number of infiltrated inflammatory cells. Similar decreases in skin thickness (−29.02% and −18.93% reduction in 1.0 and 0.3 (g/kg/day) MEAH-treated groups, resp.) and the numbers of infiltrated inflammatory cells (−61.93% and −30.14% reduction in 1.0 and 0.3 (g/kg/day) MEAH-treated groups, resp.) were also observed in *ventrum pedis* skin (Figure 7 and Table 1). Therefore, the results from the carrageenan-induced paw edema model indicate that MEAH effectively attenuated the acute phase of inflammatory swelling and infiltration of inflammatory cells.

## 4. Discussion 

*A. apiacea* Hance is distributed on river beaches of East Asia. Traditionally, *A. apiacea* Hance has the effects of removing fever from the blood and has been widely prescribed to cure human disorders including malaria, jaundice, skin disorders, and dyspeptic complaints in oriental medicine [9]. The compounds isolated from *Artemisia* species such as terpenoids, flavonoids, coumarins, acetylenes, caffeoylquinic acids, and sterol were shown to have antimalarial, antiviral, antitumor, antipyretic, antihemorrhagic, anticoagulant, antianginal, antioxidant, antihepatitis, antiulcerogenic, antispasmodic, anticomplementary, and interferon-inducing activities [14]. Among them, artemisin and its congeners have been used as standard treatment for malaria. In addition, the therapeutic potential of those compounds as anticancer, antiangiogenesis, antiviral, immunosuppressive, and antifungal agents has been extensively tested [11]. In an effort to identify active constituents of MEAH, MEAH was additionally analyzed by GC-MS. Unfortunately, we could not detect artemisin in MEAH by GC-MS analysis. Our supplementary results indicated that fatty acids and their derivatives are among the major compounds found in MEAH (see Supplementary Table 1 in Supplementary Material available online at http://dx.doi.org/10.1155/2013/494681). Interestingly, it has been reported that uniphat A60 (methyl palmitate) reduced the inflammatory phenotypes in several experimental animal models [15]. Moreover, uniphat A60 inhibited phagocytic activity and decreased the expression of proinflammatory genes through NF-*κ*B inactivation in macrophage lineage cells [16–19]. Scopoletin found in MEAH also suppressed the production of proinflammatory mediators in LPS-stimulated Raw264.7 cells [20]. Therefore, the compound (or combination of compounds) that accounts for the anti-inflammatory effect of MEAH needs to be clarified in the near future. Regardless of a number of studies about therapeutic potential of the constituents in *A. apiacea* Hance, the pharmacological effect of *A. apiacea* Hance against acute inflammation has not been fully established. As far as new pharmacological aspects of *A. apiacea* Hance, the results from this study clearly showed that MEAH inhibited the expression of proinflammatory enzymes and cytokines in LPS-stimulated Raw264.7 cells and also reduced paw edema in rats injected with carrageenan. 

Endotoxins are the prominent initiators of the pathological process of inflammation in the human body. In particular, LPS can directly activate immune cells such as macrophages, which have an important role in immune response [21]. In the progression of inflammation, NO, prostanoids, TNF-*α*, and ILs are known as the most important mediators produced by macrophages [22, 23]. Among signaling molecules associated with the production of these inflammatory mediators, iNOS and COX-2 are the most crucial enzymes involved in the production of NO and PGs [24, 25]. In this study, we verified the ability of MEAH to inhibit the production of NO and PGE_2_ in LPS-stimulated Raw264.7 macrophage cells. Treatment with MEAH effectively decreased NO and PGE_2_ production in the medium. Next, we assessed the level of iNOS and COX-2 proteins by immunoblot analyses. Pretreatment with MEAH significantly inhibited iNOS and COX-2 expression. Although MEAH markedly blocked iNOS induction, its inhibition of COX-2 was slight, but significant, showing that anti-inflammatory activity of MEAH more likely results from its inhibition of iNOS than of COX-2. 

 Among the inflammatory cytokines, TNF-*α*, IL-1*β*, and IL-6 are the most crucial mediators in the inflammatory process, mediating immunity and activating macrophages [6, 26]. TNF-*α*, the key mediator in inflammatory responses, can activate immune cells including macrophages [27]. In the process of endotoxin-induced organ injury, TNF-*α* is regarded as a principal mediator in stimulating secretion of other cytokines. IL-1*β* and IL-6 are other important inflammatory mediators that are mainly synthesized by immune cells and play a pivotal role in the acute phase of inflammation [28]. They are also mediators of the host inflammatory response in innate immunity in association with gram negative sepsis. We verified the effects of MEAH on secretion of the cytokines in LPS-stimulated Raw264.7 cells. MEAH pretreatment significantly decreased the ability of LPS to induce production of TNF-*α*, IL-1*β*, and IL-6 in the macrophages. These results showing that MEAH inhibits inflammatory cytokine production as well as iNOS and COX-2 expression indicate that MEAH has an anti-inflammation role through inactivation of macrophages. Therefore, we next examined the effect of MEAH on the representative transcription factor in association with acute inflammation. 

 The binding of LPS to the Toll-like receptor-4 recruits a variety of adaptor molecules including MyD88, TIRAP/Mal, TRIF, and TRAM and then activates the NF-*κ*B pathway for the induction of proinflammatory genes [29, 30]. After phosphorylation and subsequent degradation of I-*κ*B*α*, NF-*κ*B, which is dissociated from the NF-*κ*B/I-*κ*B complex, translocates into the nucleus and then binds to the promoter region for induction of the inflammatory response. Because NF-*κ*B binding to the promoter of iNOS, COX-2, TNF-*α*, IL-1*β* and IL-6 genes is essential for the gene induction [28, 31–34], the effect of MEAH on the NF-*κ*B signaling pathway was investigated as a plausible anti-inflammatory mechanism. The results from this study suggest that MEAH pretreatment attenuated the degradation of I-*κ*B*α*, the phosphorylation of I-*κ*B*α*, and nuclear accumulation of NF-*κ*B. 

 In the animal model of acute inflammation, the paw edema model using carrageenan is one of the most well-established models in association with edema formation [7]. This animal model is frequently used for screening of novel anti-inflammatory drugs. In the pathological process of inflammation, swelling is induced by an increase in vascular permeability and results in inflammation by leading to infiltration of inflammatory cells in the sites. Due to the importance of swelling in the inflammation process and the demand for *in vivo* verification of the MEAH effect, we confirmed the ability of MEAH to inhibit swelling of paw injected with carrageenan in rats. Carrageenan treatment significantly induced paw swelling, and administration of MEAH to rats markedly decreased the induction of paw edema. Carrageenan-induced paw swelling might be related to the release of NO in the peripheral tissue [35]. It has been also reported that carrageenan induces the release of TNF-*α* and ILs in the tissue [36]. Therefore, the inhibitory effects of MEAH on formation of swelling might, at least in part, be associated with the inhibition of NO and TNF-*α* productions.

In conclusion, the present results demonstrated that methanol extracts of *A. apiacea* Hance inhibited the production of NO through the inhibition of iNOS expression in LPS-stimulated Raw264.7 cells. In addition, MEAH attenuated the induction of COX-2 and other proinflammatory cytokines (TNF-*α*, IL-1*β*, and IL-6) by LPS. Downregulation of the NF-*κ*B signaling pathway was also involved in the decrease in proinflammatory enzymes and cytokines expressions. Furthermore, MEAH treatment decreased carrageenan-induced inflammatory swelling and the number of infiltrated inflammatory cells *in vivo*. The present findings showing the inhibition of inflammatory response both *in vitro* and *in vivo* increase our understanding of the novel pharmacologic aspects of *A. apiacea* Hance and suggest its potential as a novel therapeutic candidate for managing inflammatory disorders.

## Supplementary Material

The major compounds in MEAH were identified by GC-MS analyses. Mass spectral analyses were performed using the NIST05 library resident in the computer. The relative peak area was calculated using the area normalization method.

## Figures and Tables

**Table 1:** Changes in the histomorphometrical analysis of hind paw skins.

Groups	Thickness (epidermis to dermis, mm)	Infiltrated inflammatory cells (cells/mm^2^ of dermis)
*Dorsum pedis* skin		
Carrageenan	2.362 ± 0.168	118.60 ± 10.21
Carrageenan + dexamethasone	0.754 ± 0.082**	31.00 ± 5.96**
Carrageenan + MEAH 1.0 g/kg	1.800 ± 0.128**	43.40 ± 5.23**
Carrageenan + MEAH 0.3 g/kg	2.052 ± 0.090**	87.80 ± 10.71**

*Ventrum pedis* skin		
Carrageenan	1.268 ± 0.039	1263.60 ± 179.74
Carrageenan + dexamethasone	0.776 ± 0.048**	86.40 ± 12.70**
Carrageenan + MEAH 1.0 g/kg	0.900 ± 0.052**	481.00 ± 55.86**
Carrageenan + MEAH 0.3 g/kg	1.028 ± 0.081**	882.80 ± 109.97**

The thicknesses of *dorsum* and *ventrum  pedis* and infiltrated inflammatory cells were measured using automated image analyzer. All values are expressed as mean ± S.D. of five rat hind paws (significant as compared with carrageenan-treated group, **P < 0.01).
