# Supplementary material for: Methanol Extract of Artemisia apiacea Hance Attenuates the Expression of Inflammatory Mediators via NF-κB Inactivation
Source: Evid Based Complement Alternat Med. 2013 Oct 22;2013:494681. doi: 10.1155/2013/494681 (PMC3819789; doi:10.1155/2013/494681)
Supplement: Supplementary file 1 — The major compounds in MEAH were identified by GC-MS analyses. Mass spectral analyses were performed using the NIST05 library resident in the computer. The relative peak area was calculated using the area normalization method. [file 494681.f1.docx]

**Supplementary Materials and Methods**

*GC-MS analysis of MEAH.* Chemical composition of MEAH was interfaced to an Agilent 6890 GC coupled to an Agilent 5975C MS operated in electron impact ionization (EI) mode (70 eV). Chromatographic separation was performed on a 30 m length × 0.25 mm i.d. and 0.25 μm film thickness fused silica capillary column HP-5MS supplied by Agilent. Helium was used as column carrier gas at a constant flow rate of 1.0 mL/min and the splitless injector temperature was set as 280°C. The column temperature program was as follows: initial temperature of 70°C for 4 min, and increased by 2°C/min 70 to 100°C (held 2 min), After that the temperature was varied from 100 to 200°C at 5°C/min (held 20 min), increase to 280°C (held 5 min) at 10°C /min, in a total run time of 120 min (Supplementary table 1).

**Supplementary table 1: Chemical compounds identified by GC-MS analysis in MEAH.**

| No. | Compound | Retention Time (min) | | Peak area (%) |
| --- | --- | --- | --- | --- |
| 1 | Dimethylphosphine | | 2.142 | 30.08 |
| 2 | 2(3H)-Furanone | | 9.494 | 0.41 |
| 3 | 1,2,4-Benzentriol | | 12.979 | 0.86 |
| 4 | Isoborneol | | 14.203 | 0.94 |
| 5 | Bicyclo[2,2,1]heptan-2-one | | 14.581 | 0.22 |
| 6 | 2-Furancarboxaldehyde | | 22.186 | 0.60 |
| 7 | Benzisothiadiazole | | 26.963 | 0.99 |
| 8 | DL-proline | | 27.593 | 0.44 |
| 9 | Nonanedioic acid | | 29.653 | 0.74 |
| 10 | Myristic acid | | 30.980 | 0.93 |
| 11 | Uniphat A60 | | 33.973 | 27.28 |
| 12 | Palmitic acid | | 35.146 | 5.57 |
| 13 | 2,4-Dihydroxy-6-methoxy-acetophenone | | 35.953 | 1.01 |
| 14 | Methyl oleate | | 37.698 | 3.12 |
| 15 | Methyl linoleate | | 38.013 | 3.01 |
| 16 | Heptadecene-(8)-carbonic acid | | 38.842 | 2.49 |
| 17 | 9,12-Octadecanoic acid | | 39.237 | 2.96 |
| 18 | Scopoletin | | 42.035 | 1.34 |
| 19 | Clionasterol | | 98.688 | 1.29 |
